# Structural and functional brain scans from the cross-sectional Southwest University adult lifespan dataset

**DOI:** 10.1038/sdata.2018.134

**Published:** 2018-07-17

**Authors:** Dongtao Wei, Kaixiang Zhuang, Lei Ai, Qunlin Chen, Wenjing Yang, Wei Liu, Kangcheng Wang, Jiangzhou Sun, Jiang Qiu

**Affiliations:** 1 Key Laboratory of Cognition and Personality, Ministry of Education, Chongqing 400715, China; 2 Faculty of Psychology, Southwest University, Chongqing 400715, China; 3 Center for the Developing Brain, Child Mind Institute, New York, NY 10022, USA; 4 Donders Institute for Brain, Cognition, and Behaviour, Radboud University Medical Centre, Nijmegen 6525 EZ, The Netherlands

**Keywords:** Ageing, Neuroscience

## Abstract

Recently, the field of developmental neuroscience has aimed to uncover the developmental trajectory of the human brain and to understand the changes that occur as a function of ageing. Here, we present a dataset of functional magnetic resonance imaging (fMRI) data covering the adult lifespan that includes structural MRI and resting-state functional MRI. Four hundred ninety-four healthy adults (age range: 19-80 years; Males=187) were recruited and completed two multi-modal MRI scan sessions at the Brain Imaging Center of Southwest University, Chongqing, China. The goals of the dataset are to give researchers the opportunity to map the developmental trajectories of structural and functional changes in the human brain and to replicate previous findings.

## Background & Summary

Magnetic resonance imaging (MRI) has become one of the most dominant techniques to investigate the human brain because it permits detailed, noninvasive and safe assessments of the human brain. MRI is also able to perform data collection using various imaging modalities, such as structural magnetic resonance imaging (sMRI), functional MRI (fMRI) and diffusion tensor imaging (DTI). In particular, these imaging measurements have been effectively used to capture structural and functional brain changes during development^1^, ageing^2^, psychiatric disorders^3^, etc. For example, the feature of resting-state functional connectivity can predict the maturity of the individual across development^4^ and can be used as a “fingerprint” to identify individuals^5^. Thus, the measurements of MRI have made great contributions as imaging biomarkers of normal development, ageing, clinical diagnosis and therapeutic assessments.

One of the most urgent scientific issues confronting us in the 21^st^ century is how we can maintain a healthy mind for human life. In addition to focusing on uncovering the developmental course by which an original brain transforms into a mature one, another critical question in lifespan developmental neuroscience is how the brain changes as a function of ageing. There is a necessity to answer this question because we can only reveal the mechanism underlying healthy brain ageing if we can discover the causes of brain diseases related to ageing (e.g., Alzheimer’s disease). Based on the measurements from various image modalities, researchers have uncovered many appealing findings in the normal ageing brain. For example, most brain regions follow a linear decline in grey matter volume (GMV) with normal ageing, while nonlinear age trajectories have also been observed in some regions (e.g., the medial temporal lobe), which indicated a preservation of GMV during the early adult lifespan^6^; increasing age was found to be accompanied by decreasing functional segregation of brain systems, and this age-related effect was more prominent in associative systems than in sensory and motor systems^7^.

For the sake of characterizing age-related changes in cognition, as well as in brain structure and function, the data repository for the Cambridge Centre for Ageing and Neuroscience (Cam-CAN) initial study cohort has provided a multimodal dataset from a large, cross-sectional adult lifespan population-based sample^8^. In addition, there are a number of datasets that are publicly available for free for authorized investigators, such as the Open Access Series of Imaging Studies (OASIS) and Alzheimer’s Disease Neuroimaging Initiative (ADNI, (http://adni.loni.usc.edu/data-samples/) datasets. OASIS consists of a cross-sectional collection of 416 subjects aged 18 to 96 and a longitudinal MRI Data collection from 150 subjects aged 60 to 96 (http://www.oasis-brains.org/). However, there is still a lack of open access datasets that expand beyond the Caucasian white population, which would allow researchers to discover meaningful regulators of normal brain ageing or to verify previous findings. Moreover, revelations of age-related changes in the human brain should be based on large continuous samples, which, in a way, limit research activities in ageing. Thus, an additional open access normal adult lifespan dataset consisting of a large sample is needed for researchers who are interested in this domain or who require an independent dataset for cross-validation. Here, we describe the data generated in the Southwest University Adult Lifespan Dataset (SALD), which is one part of our ongoing project to examine the associations among brain imaging, creativity and mental health (BCM). The SALD comprises a large cross-sectional sample (total scans=494; age span=19-80 years) and a multi-modal (sMRI and rs-fMRI) investigation of these neural underpinnings. The goal of the SALD is to understand what a normal brain looks like and how it structurally and functionally changes at each decade of life from age 20 through age 80. Currently, it is available for research through the International Data-sharing Initiative. We hope our free data sharing can hasten the progress of normal brain ageing studies.

## Methods

### Participants

**Figure 1:**
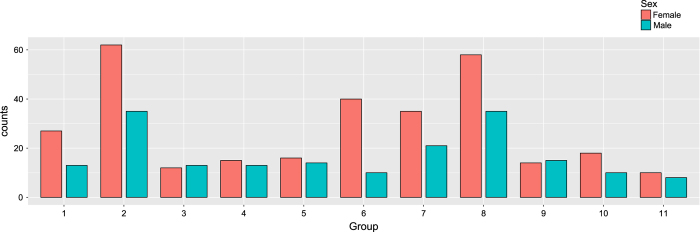
The distribution of participants based on age and gender. Participants were separated into 11 groups based on their age and gender respectively. The x-axis indicates the age group, and the y-axis indicates the number of participants. The blue bar indicates male participants, and the red bar indicates female participants; in addition, the exact numbers of them are shown on the corresponding bar. Note that, for a relatively balanced distribution, the age span was set as 4 years in the first two groups, and 6 years in the remaining groups.

The 494 participants (308 Females, 187 Males, aged 19 to 80 years) included in the release were selected from a large dataset of individuals who participated in the ongoing BCM data collection initiative. The young adults (18–25) of the lifespan sample were enrolled as college students at Southwest University in Chongqing, China. The university enrols 10,000 ordinary undergraduates each year, nearly 65% of who are female. The young adults were collected by random sampling from Southwest University; thus, the number of young females (20–27) is larger in our dataset (for more details, see Fig. 1). Many of the middle-aged adults (age 26 to 40 years) were recruited directly from staff at Southwest University. The rest of the participants in the adult sample were recruited from communities close to the university campus. The data collection was initiated in 2010 and was terminated in 2015. In addition, a part of the participants served as a control sample in a case-controlled study of a clinical population. Some participants were excluded due to sleeping during scanning. We primarily recruited participants through leaflets, online advertisements, and face-to-face propaganda. The exclusion criteria included the following: (1) MRI-related exclusion criteria, which included claustrophobia, metallic implants, Meniere’s Syndrome and a history of fainting within the previous 6 months; (2) current psychiatric disorders or neurological disorders; (3) use of psychiatric drugs within the three months prior to scanning; (4) pregnancy; or (5) a history of head trauma. Informed written consent was obtained from each participant. In addition, we required the participants to refrain from drinking the day before the scanning and the day of the scanning. The dataset collection was approved by the Research Ethics Committee of the Brain Imaging Center of Southwest University, in accordance with the Declaration of Helsinki. Written informed consent was obtained from all participants prior to the data collection.

### Image Acquisitions

All of the data were collected at the Southwest University Center for Brain Imaging using a 3.0-T Siemens Trio MRI scanner (Siemens Medical, Erlangen, Germany). Each participant took part in 3D structural MRI and resting-state fMRI scans; only subgroups of the participants underwent a task-based fMRI scan (the task data are not part of this release). Additionally, to avoid the lasting effect of task-based fMRI on the resting-state fMRI, the resting-state scan was performed before a particular task. For each participant, the 3D structural MRI and resting-state sequences were acquired in succession within one session. The anatomical and resting-state date was collected with the following parameters.

#### (1) 3D structural MRI

A magnetization-prepared rapid gradient echo (MPRAGE) sequence was used to acquire high-resolution T1-weighted anatomical images (repetition time=1,900 ms, echo time=2.52 ms, inversion time=900 ms, flip angle=90 degrees, resolution matrix=256×256, slices=176, thickness =1.0 mm, and voxel size=1×1×1 mm^3^).

#### (2) Resting-state fMRI

During the resting-state MRI scan, the subjects were instructed to lie down, close their eyes, and rest without thinking about any specific thing but to refrain from falling asleep. The 8- min scan of 242 contiguous whole-brain resting-state functional images was obtained using gradient echo echo-planar-imaging (GRE-EPI) sequences with the following parameters: slices=32, repetition time (TR)/echo time (TE)=2000/30 ms, flip angle=90, field of view (FOV)=220×220 mm, thickness/slice gap=3/1 mm, and voxel size=3.4×3.4×4 mm^3^.

### Code availability

We shared the code we used in the quality assessment (QA), voxel-based volume and functional connectivity analyses, and it is freely available on GitHub (https://github.com/Zhuang2KX/SALD).

## Data Records

This dataset is publicly available through the International Data-sharing Initiative (Data Citation 1). We removed the facial information of each participant from the sMRI data (https://github.com/poldracklab/pydeface) and the Neuroimaging Informatics Technology Initiative (NIFTI) headers according to FCP/INDI policies. The contents and data structures of these packages are detailed as follows:

### MRI data and demographic information

Location:

sMRI

RawData_BIDS/sub-*/anat/

rsfMRI

RawData_BIDS/sub-*/func/

All the imaging data are organized according to the BIDS criteria^9^. For more detailed information, please visit the following website: http://bids.neuroimaging.io/.

Basic phenotypic information

File format: ‘sub_information.xlsx’ files

Basic demographic information including age, sex and handedness is provided in the ‘sub_information.xlsx’ file. In addition, the quality assessment measures of the different scans were also included in this file.

### Quality Control Report

The folder quality-assessment-protocol compackage contains the quality assessment (QA) analysis results performed in the present study for the structural and functional images. It contains ‘sub_information.xlsx’ files (QC_anatomical_spatial, QC_functional_spatia.csv, and QC_functional_temporal, respectively). Those files were generated by the Preprocessed Connectomes Project (PCP) quality assessment protocol, and we did not change any part of the pipeline. For more details about its procedure and the measures included, see the website for the PCP quality assessment protocol (http://preprocessed-connectomes-project.org/quality-assessment-protocol/). All data were made available to users regardless of the data quality because there were no consensus criteria by which to determine what kind of MRI images should be excluded.

## Technical Validation

### Results of QA measures

**Figure 2:**
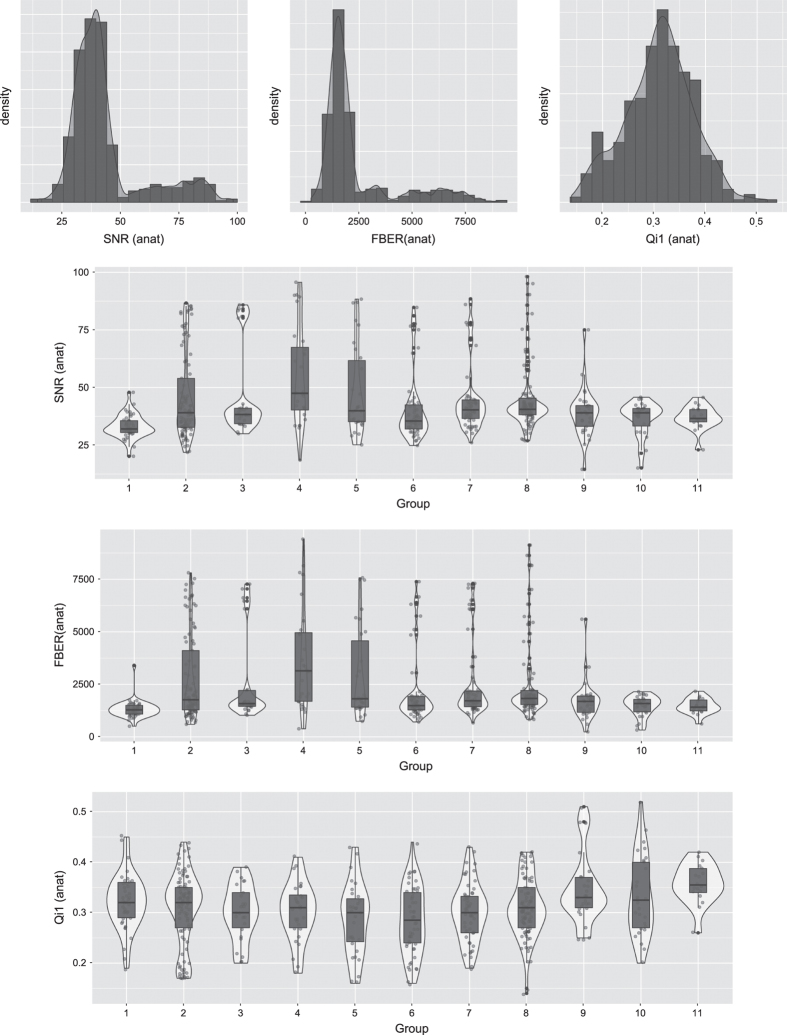
The distributions of several representative QA measures of the structural MRI data across all participants. SNR is the abbreviation for the signal-to-noise ratio. This ratio indicates the mean intensity within the grey matter divided by the standard deviation of the values outside the brain. Higher values are better. FBER is the abbreviation for the foreground to background energy ratio. This ratio indicates the variance among the voxels inside the brain divided by the variance among the voxels outside the brain. Higher values are better. Qi1 means the percent of artefact voxels, which implies the proportion of voxels outside the brain with artefacts to the total number of voxels outside the brain. Lower values are better.

**Figure 3:**
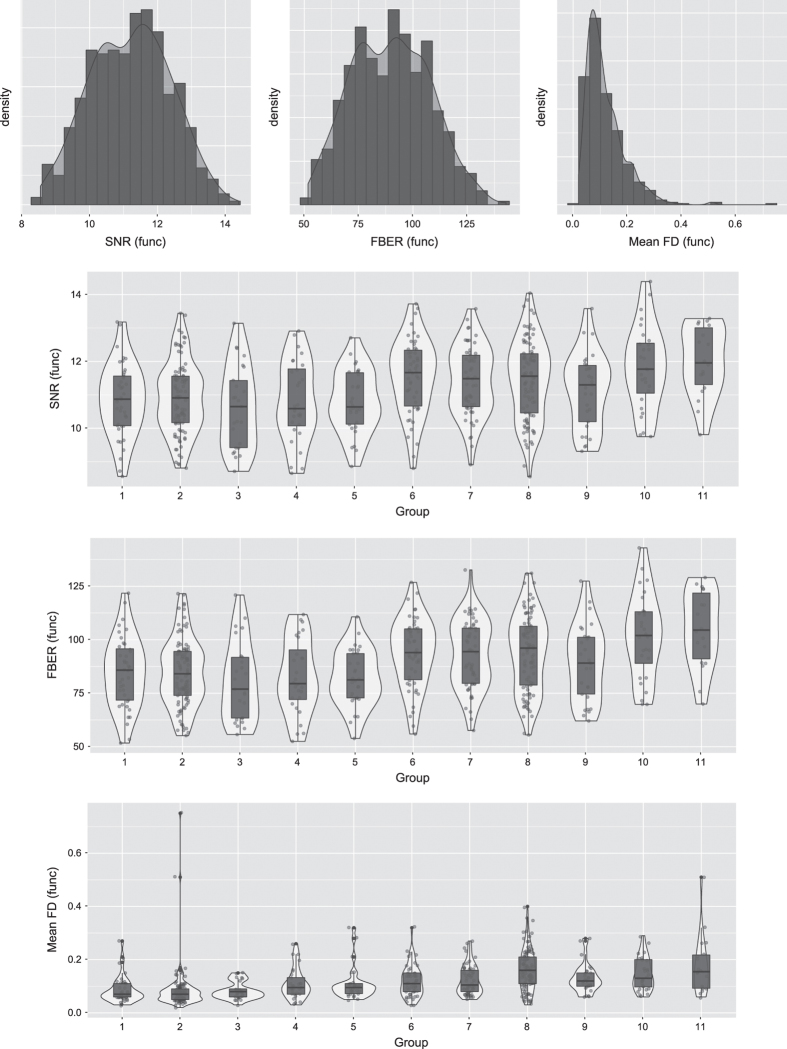
The distributions of several representative QA measures of the resting-state fMRI data across the participants. SNR is the abbreviation for the signal-to-noise ratio. This ratio indicates the mean intensity within the grey matter divided by the standard deviation of the values outside the brain. Higher values are better. FBER is the abbreviation for the foreground to background energy ratio. This ratio indicates the variance among the voxels inside the brain divided by the variance among the voxels outside the brain. Higher values are better. Mean FD refers to the mean fractional displacement-Jenkinson. It is a measure of subject head motion that compares the motion between the current and previous volumes. Mean FD is calculated by summing the absolute value of the displacement changes in the x, y and z directions and rotational changes about those three axes. The rotational changes are given as distance values based on the changes across the surface of a sphere with a radius of 80 mm. Lower values are better.

To quantitatively assess the quality of the MRI data, a series of widely used QA measures were calculated. All measures computed by the PCP quality assessment protocol can be found together with the data. Figures. 2 and 3 indicate the distributions of several representative QA measures of the structural MRI and resting-state fMRI data across participants, respectively. For more information about the QA measures, see the uploaded ‘sub_information.xlsx’ files.

### Relationship among age, head motion and signal-to-noise ratio (SNR)

**Figure 4:**
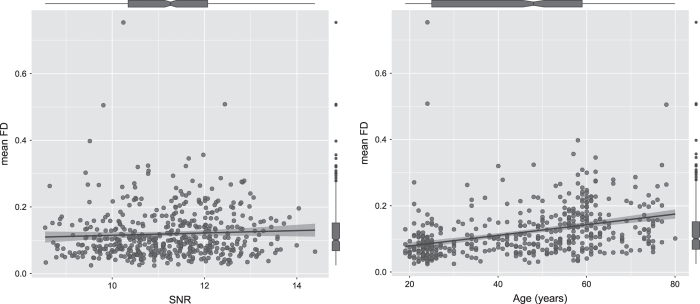
The effects of the mean FD on age and SNR. The x-axes indicate age and SNR values, respectively. The y-axis indicates the mean FD values.

To investigate the impact of head motion during the resting-state fMRI scanning on the overall quality of images and its association with age, we correlated the head motion (as measured by the mean framewise displacement) with both age and the SNR of the entire sample (*N*=494). The results revealed that no significant result was found in the relationship between mean framewise displacement (FD) and SNR (*r*=0.055, *P*=0.222). There was a significant and positive correlation that existed between the mean FD and age (*r*=0.372, *P*<0.001), and this relationship strengthened (*r*=0.455, *P*<0.001) after we removed 16 subjects who possessed the outlying mean FD values. Figure 4 indicates these two correlations. The results suggested that head motion may increase with age, and the head motion in this dataset did not significantly affect the overall quality of the images in a linear trend.

### Replication of previous findings

To test whether this dataset was technically valid, we tried to use the current data to replicate some previous findings. Here, the sMRI and resting-state fMRI data were analysed based on this objective.

### 3D structural MRI data

A large number of studies have reported that structural development during normal ageing is accompanied by a decline in the total grey matter volume^10–13^; in addition, cortical grey matter volume was found to decline throughout adulthood^14–16^. In addition, the decreases were always reported to be most pronounced in the frontal and parietal lobes^6,14,17,18^. Here, we attempted to replicate these robust findings in the current dataset.

The sMRI (1×1×1 mm^3^) data were preprocessed using SPM8 (Wellcome Department of Cognitive Neurology, London, UK; www.fil.ion.ucl.ac.uk/spm). For better registration, all T1-weighted structural images were automatically co-registered to the anterior commissure-posterior commissure (AC-PC) using an SPM8-based script. Then, a spatially adaptive non-local means (SANLM) denoising filter^19^ was applied using the VBM8 toolbox (http://www.neuro.uni-jena.de/vbm/download/). Next, using the unified segmentation procedure, the co-registered images from each participant were segmented into grey matter (GM), white matter and cerebrospinal fluid^20^. The GM images of each participant were spatially normalized to a study-specific T1-weighted template using a diffeomorphic nonlinear registration algorithm (DARTEL; diffeomorphic anatomical registration through exponentiated lie algebra). The DARTEL registration involves the following: first, computing the specific template based on the average tissue probability maps from all the participants; and second, warping each participant’s segmented maps to a specific template. To improve the alignment and to achieve a more accurate inter-subject registration, the procedure was repetitively conducted until the best study-specific template was generated. Subsequently, registered images were transformed to the Montreal Neurological Institute (MNI) space, and a further modulation was conducted to preserve the volume of the GM. Finally, a 6-mm full width at half maximum (FWHM) Gaussian kernel was applied to smooth the modulated GM images.

We first used Pearson correlation to detect the relationship between age and total grey matter volume (GMV). Then, multiple linear regression analyses were used to determine GMV regions that were associated with age, controlling for the total GMV. To avoid edge effects around the borders between the GM and WM, we used explicit masking to restrict the search volume. The explicit masking was achieved using the SPM masking toolbox (http://www.cs.ucl.ac.uk/staff/g.ridgway/masking/). This approach reduced the risk of false negatives caused by overly restrictive masking, as potentially interesting voxels may be excluded from the statistical analysis^21^. For the regression analysis, we used the family-wise error (FWE) of *P*<0.05 at the whole-brain level and≥20 contiguous voxels as a threshold to correct for multiple comparisons.

**Figure 5:**
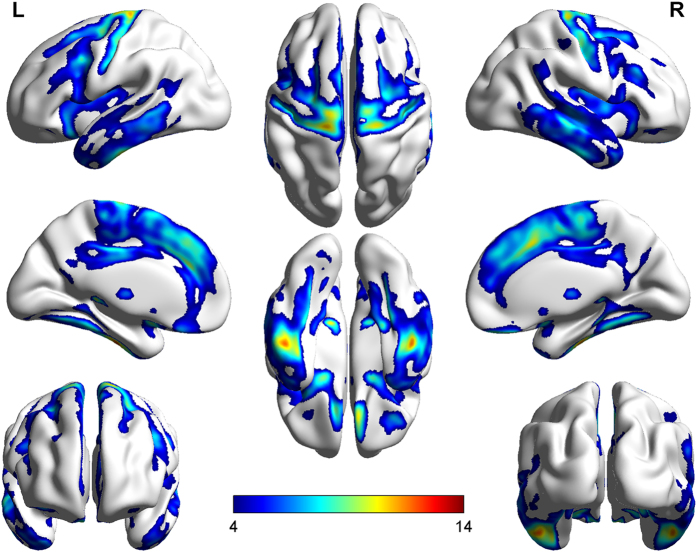
Brain regions with GMV reductions during normal ageing. L-R means from the left hemisphere of the brain to the right hemisphere.

The results indicated that age was significantly correlated with total GMV (*r*=−0.305, *P*<0.001). Almost all areas of the cerebral cortex exhibited a significant age-related decline in GMV. In addition, the frontal, parietal and temporal lobes showed the most pronounced function, which to a large extent confirmed the previous findings (Fig. 5). However, in accordance with one prior study^6^, we found that the occipital regions were less affected by age.

### Resting-state fMRI data

There is a widely reported finding indicating that clear segmentation between neural systems would consistently become lost over the course of normal human ageing; many intrinsic functional connectivity brain networks gradually become less internally coherent with age^7,22–24^. In an attempt to replicate this finding, the current dataset was used to describe the changing trajectories of within-system connectivity along with age.

The resting-state fMRI data were preprocessed using Data Processing Assistant for Resting-State fMRI (DPARSF_V4.2, http://resting-fmri.sourceforge.net/), implemented on the MATLAB 2014a (Math Works, Natick, MA, USA) platform. The first 10 volumes of the functional images were discarded to account for signal equilibrium and the participants' adaptations to their immediate environment. The remaining 232 scans were corrected for slice timing and were then realigned to the middle volume to correct for head motion. Participants with head motion exceeding 2.0 mm in any dimension throughout the course of the scans were discarded from further analysis. Subsequently, the registered images were spatially normalized to the Montreal Neurological Institute (MNI) template (resampling voxel size=3×3×4 mm^3^). Next, nuisance signals representing motion parameters, global signals, white matter, and cerebrospinal fluid signals were regressed out in order to control the potential impact of physiological artefacts. Here, we used the Friston 24-parameter model, which includes 6 motion parameters, 6 temporal derivatives, and their squares^25,26^ to regress out head motion effects. This approach was based on recent research that demonstrated that higher-order models are more effective at reducing the effects of head movements^27–29^. Then, after the spatial smoothing (full width at half maximum=6 mm Gaussian kernel), bandpass filtering (0.009–0.08 Hz) was performed. These preprocessing steps followed the standard protocol published^28^.

Whole-brain functional connectomes were constructed for each subject as a 264×264-node graph, labelled by functional systems^27^. Edge weights were calculated as the Fisher z-transformed correlation (Pearson’s r) between each pair of nodes, and negatively weighted edges were removed from each correlation matrix to eliminate potential misinterpretation of the negative edge weights. For each specific system, the within-system connectivity was calculated as the mean node-to-node z-value of all nodes of that system to each other. The mean within-system connectivity means the average value of within-system connectivity over all of the systems.

**Figure 6:**
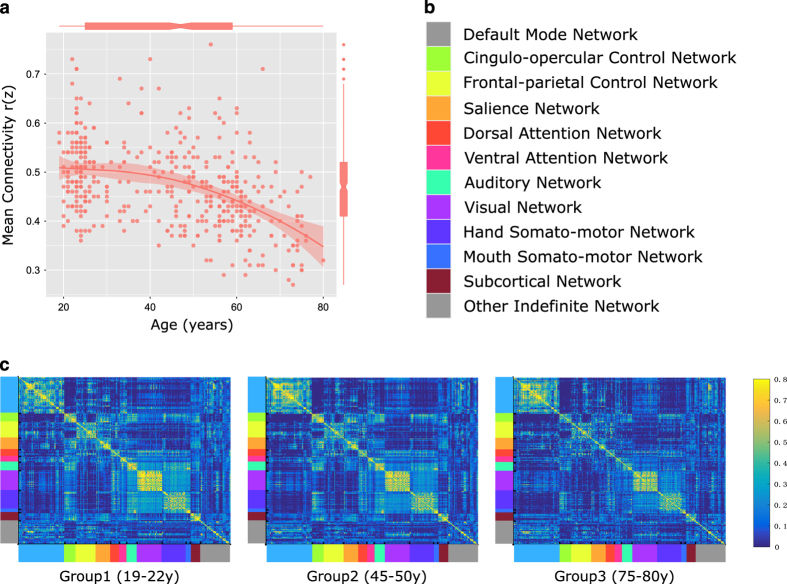
Within-system connectivity decline with age. (**a**) Demonstrates the negative correlation between age and mean connectivity. (**b**) Displays the different brain networks involved in this analysis. The mean connectivity in (**a**) was calculated by averaging the intrinsic functional connectivity within each of the networks. (**c**) Displays the functional connectivity matrices of three representative age groups. The networks were arranged in the same order as **b**. It can be seen that the within-system connectivity apparently declined with age.

The results indicated that the mean within-system connectivity would decrease with age. When we applied linear and nonlinear (second-degree polynomial) models to the within-system connectivity, we found that the age function was fit significantly both by the linear model (adjusted *R*^*2*^=0.177, *P*<0.001) and by the quadratic model (adjusted *R*^*2*^=0.195, *P*<0.001). There is a significant difference in two models (F=10.9, *P*<0.001). The quadratic regression model has a smaller Akaike Information Criterion (AIC=−1010.18) compared to linear model (AIC=−1001.24). If models that included more parameters than a linear parameter reduced AIC by at least 2.5 points, the more complex model was selected as a better fit to the data^30^. The figure shows a decrease (probably non linear) of functional connectivity with age (Fig. 6).

## Usage Notes

We encourage other labs to use the dataset described in this publication under the requirement of citing the present data descriptor. Data for the SALD are available for download in an Amazon Web Services S3 bucket from the International Data-sharing Initiative (http://fcon_1000.projects.nitrc.org/indi/s3/index.html). The results of the quality analysis measurements are available for free to download and use according to the consensus criteria to determine what kind of MRI images should be excluded. We hope that all users of the data will acknowledge the original authors by citing this publication.

## Additional information

**How to cite this article**: Wei, D. *et al*. Structural and functional brain scans from the cross-sectional Southwest University adult lifespan dataset. *Sci. Data* 5:180134 doi: 10.1038/sdata.2018.134 (2018).

**Publisher’s note**: Springer Nature remains neutral with regard to jurisdictional claims in published maps and institutional affiliations.

## Supplementary Material
